# A Study on Multi-Robot Task Allocation in Railway Scenarios Based on the Improved NSGA-II Algorithm

**DOI:** 10.3390/s25041001

**Published:** 2025-02-07

**Authors:** Yanni Shen, Jianjun Meng

**Affiliations:** 1 School of Mechanical Engineering, Lanzhou Jiaotong University, Lanzhou 730070, China; 13220039@stu.lzjtu.edu.cn; 2 Institute of Mechanical and Electrical Technology, Lanzhou Jiaotong University, Lanzhou 730070, China; 3 Gansu Engineering Technology Research Center of Logistics and Transportation Equipment Informatization, Lanzhou 730070, China; 4 Gansu Logistics and Transportation Equipment Industry Technology Center, Lanzhou 730070, China

**Keywords:** multi-robot systems, railway multi-robot task allocation, multi-objective optimization, NSGA-II, MOPSO

## Abstract

With the advent of Industry 4.0, the seamless integration of industrial systems and unmanned technologies has significantly accelerated the development of smart industries. However, the research on task allocation for railway maintenance robots remains limited, particularly with respect to optimizing costs and efficiency within smart railway systems. To address this gap, the present study explores multi-robot task allocation for automated orbital bolt maintenance, aiming to enhance operational efficiency by minimizing both makespan and total travel distance for all robots. To achieve this, an improved hybrid algorithm combining NSGA-II and MOPSO is proposed. Initially, a dynamic task planning method, tailored to the specific conditions of railway operations, is developed. This method uses the coordinates of track bolts to extract environmental features, enabling the dynamic partitioning of task areas. Subsequently, a multi-elite archive strategy is introduced, along with an adaptive mechanism for adjusting crossover and mutation probabilities. This ensures the preservation and maintenance of multiple solutions across various Pareto fronts, effectively mitigating the premature convergence commonly observed in traditional NSGA-II algorithms. Moreover, the integration of the MOPSO algorithm strikes a balance between local and global search capabilities, thereby enhancing both optimization efficiency and solution quality. Finally, a series of experiments, conducted with varying task sizes and robot quantities during the railway maintenance window, validate the effectiveness and improved performance of the proposed algorithm in addressing the multi-robot task allocation problem.

## 1. Introduction

**Figure 1:**
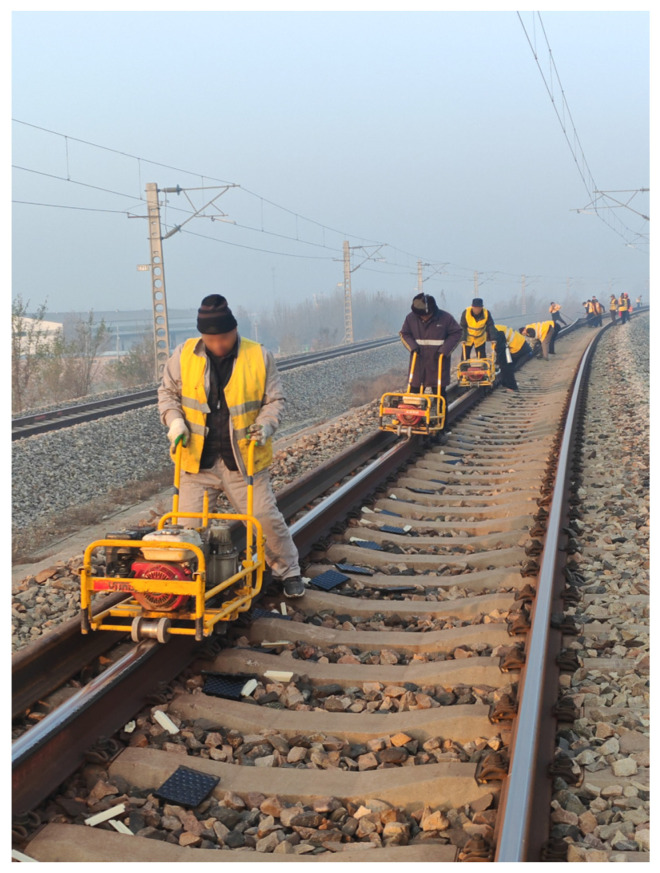
Railway maintenance personnel operating during the window period.

In the context of the rapid development of Industry 4.0, unmanned vehicles and robotic technologies have increasingly permeated diverse industrial domains, encompassing manufacturing, warehousing and logistics, agriculture, and healthcare [1,2,3,4]. Harnessing automation and intelligent technologies, these unmanned systems are capable of executing tasks proficiently and precisely, minimizing human errors and mitigating safety hazards. Fasteners, as a crucial element of railway track infrastructure, directly influence the operational safety of trains and the stability of the track system [5,6]. Adequate maintenance of track fasteners not only contributes to reducing vibrations and noise produced during train operations but also prolongs the service life of the track. As the demand for railway operations persists in growing, traditional maintenance methods during scheduled window periods have gradually unveiled shortcomings, such as inefficiency, high labor intensity, and insufficient safety, rendering it increasingly arduous to meet the escalating demands of track maintenance. A typical window period maintenance situation in a railway maintenance section is illustrated in Figure 1. Particularly, the maintenance of track bolts poses substantial challenges owing to the complexity of the task and the considerable quantity of bolts concerned. High-intensity manual operations carried out under adverse weather conditions not only lead to worker fatigue but also augment the risk of accidents. Furthermore, with the increasing frequency of train operations, railway maintenance tasks must be accomplished within the limited window periods, further intensifying the pressure on traditional manual maintenance approaches. Hence, there exists an urgent necessity for more efficient and safer intelligent equipment to enhance maintenance capabilities. The research and development of the automated orbital bolt robot (AOBR) for track fastener maintenance presents a key solution to this issue and is anticipated to achieve the intelligent and automated maintenance of track fasteners, thereby significantly enhancing maintenance efficiency.

The AOBR constitutes an extremely intelligent and flexible track fastener maintenance device, capable of independently carrying out tasks such as tightening, loosening, and inspecting track bolts, thereby significantly enhancing operational efficiency and minimizing manual intervention. In comparison with traditional manual maintenance approaches, the introduction of AOBR confers several advantages. Firstly, it effectively curtails maintenance time, enhances task precision, and minimizes the risk of operational errors. Secondly, through collaborative operation among multiple AOBRs, maintenance efficiency can be further augmented, reducing the requisite window period and ensuring the smooth operation of railway transportation. Additionally, the high efficiency of AOBRs contributes to the optimized allocation of railway resources, facilitating the development of a high-quality railway service system. However, as multiple AOBRs operate simultaneously within the same work area, issues related to task interference and conflicts have gradually emerged. In track bolt maintenance, precise task allocation and coordination among different AOBRs are indispensable to avoid conflicts and maximize operational efficiency. At present, the research on task allocation for railway maintenance robots remains relatively scarce.

Consequently, the development of an efficacious task allocation algorithm and efficient coordination control strategy has become a crucial research field for the AOBR system. The fundamental objective of this paper resides in resolving the task allocation conundrum of AOBRs via multi-robot collaboration for the execution of track bolt maintenance tasks. The multi-robot task allocation matter in automated track bolt maintenance is inherently NP-hard, thereby rendering it difficult to acquire an optimum solution within a rational time period through the application of precise algorithms [7]. Heuristic methods provide an effective approach for obtaining optimal or near-optimal solutions to NP-hard problems. Among these, genetic algorithms have demonstrated strong global search capabilities in discrete optimization problems. Given that this study involves a multi-objective optimization problem, the NSGA-II algorithm has been chosen to tackle the multi-robot task allocation challenge. To adapt the algorithm to the specific characteristics of this problem, several modifications have been made, resulting in a hybrid methodology that combines the enhanced NSGA-II with multi-objective particle swarm optimization (MOPSO). The major contributions of this paper are presented as follows:

1. A mathematical model is developed for the proposed multi-robot task allocation problem, and a composite algorithm integrating the enhanced NSGA-II and MOPSO is designed to address this challenge.
2. In response to the dispersed nature of railway track fastener maintenance areas, a dynamic task planning approach tailored to the railway environment is introduced. By extracting track bolt coordinates to capture environmental characteristics, the maintenance area is dynamically partitioned. A heuristic algorithm is then applied to allocate tasks within these partitioned regions.
3. To accommodate the unique distribution of track bolts, a sequence encoding method with separators is employed to represent the task execution sequence and the division of tasks among multiple robots, thereby enhancing the clarity and operability of the task allocation process.
4. A multi-elite archive strategy is implemented, along with an adaptive mechanism to adjust crossover and mutation probabilities, ensuring the preservation and management of multiple solutions across various Pareto fronts. This approach helps to mitigate the premature convergence commonly encountered in the NSGA-II algorithm. Additionally, by incorporating the MOPSO algorithm, the hybrid approach effectively maintains a balance between local and global search capabilities, thus improving both the optimization efficiency and the quality of the resulting solutions.

The structure of this paper is organized as follows: Section 2 provides a review of the relevant literature. Section 3 presents the problem description and its mathematical formulation. Section 4 outlines the complete procedure of the hybrid algorithm. Section 5 presents the experimental results and evaluation of the algorithm. Finally, Section 6 concludes this paper and discusses potential directions for future research.

## 2. Related Work

Multi-robot task allocation, which is recognized as a fundamental NP-hard matter, has commanded considerable attention in the academic research. Additionally, the matter of multi-robot task allocation is typically defined as a multi-objective optimization problem accompanied by constraints instead of a single-objective one [8]. Researchers from both domestic and international institutions have proposed a variety of solutions to this problem, achieving significant outcomes. The main research methods include model construction based on graph theory [9], optimization using integer programming methods [10], and the application of various heuristic algorithms [11,12].

Minglei Xiong and Guangming Xie [13] integrated game theory into the task allocation and decision-making processes of underwater robot swarms operating in complex environments. Through interaction with local state information, they accomplished a combination of selfishness and altruism to maximize individual benefits, thereby facilitating task allocation that is distributed and self-organized in multi-robot systems operating in dynamic and uncertain circumstances. Li Huang et al. [14] put forward an optimization strategy for the multi-robot task allocation (MRTA) problem, offering multiple solutions to ensure the consistency of task allocation quality. To shorten the fitness evaluation time and enhance computational efficiency, they devised the sNIOA algorithm, which employs a regression model to pre-evaluate the new population, enabling the early elimination of non-ideal antibodies and thereby avoiding unnecessary computations. Subsequently, they introduced a base pair-inspired GM operator, which divides antibody genes into multiple genomes. During genetic mutation, this operator increases the probability of mutation of other genes within the same genome, facilitating the identification of better solutions within a limited number of iterations. Zhenhua Miao et al. [15] introduced an improved hybrid approach that combines a simulated annealing-based multimodal multi-objective differential evolution algorithm (IMMODE-SA), aiming to enhance the reliability and feasibility of solutions for the MRTA problem. The experimental outcomes reveal that this approach exhibits robust global search and local exploration abilities. Changyun Wei et al. [11] addressed the shortcomings of the traditional particle swarm optimization (PSO) method in directly solving multi-objective problems by proposing a new multi-objective particle swarm optimization algorithm. By utilizing a strategy for Pareto front refinement and a technique of leader selection relying on probability, this approach efficiently produces solutions for problems of multi-objective optimization. Reza Javanmard Alitappeh and Kossar Jeddisaravi [16] partitioned the map into regions in light of the distribution of task points and integrated metaheuristic methods with Q-learning to allocate tasks to each robot. Lixuan Zhang et al. [17] put forward a batch processing algorithm for the TWPC-MRTA problem. Experimental findings indicate that this methodology performs proficiently in clustering and efficient task allocation. Fuhan Yan and Kai Di [18] introduced an innovative hyper-heuristic algorithm for the MRTA problem, which optimizes the crucial parameters of the influence diffusion model by employing low-level heuristic (LLH) and particle swarm optimization (PSO) techniques. M. Rohini et al. [19] utilized genetic algorithms to address the industrial real-time MRTA problem, thereby enhancing both accuracy and performance. Shengli Wang et al. [20] presented a multi-objective ant colony optimization technique for solving the problem of cooperative task allocation in multi-robot systems. By incorporating a Pareto front-based collaborative task allocation strategy, they enhanced the algorithm’s convergence performance. Wang Yafei and Liang Zhang [21] applied a multi-objective particle swarm optimization algorithm based on adaptive region division to address the multi-UAV task allocation problem. This approach alleviates the constraints of conventional optimization algorithms that have a propensity for being trapped in local optima and presents enhanced convergence and solution diversity. Murugappan Elango et al. [22] combined K-means clustering with an auction-based mechanism to address the multi-robot task allocation issue, thereby enhancing the degree of efficiency and flexibility of the algorithm.

As an intelligent heuristic algorithm, NSGA-II typically confronts problems such as premature convergence and entrapment in local optimums during practical applications in the real world, consequently leading to suboptimal solutions within the constrained time frame. To confront these challenges, researchers have carried out diverse modifications to specific components of the NSGA-II algorithm in an attempt to attain a solution that is globally optimal.

Min Zhang et al. [23] presented a normal distribution crossover operator, which boosts the algorithm’s capacity to evade local optima. Xuyang Tian et al. [24] refined the NSGA-II algorithm by integrating a probability parameter into the binary tournament selection mechanism to govern the selection of inferior individuals, thereby ensuring population diversity in the early stages. They also adopted an adaptive crossover operator, which enlarges the search space in the initial phase and accelerates the search speed in the later stages. LI H. F. et al. [25] enhanced the update of the population mechanism of the NSGA-II algorithm by introducing a novel ranking criterion for assessing the dominance of individuals within the population. They embraced a pliable strategy to preclude the algorithm from being ensnared in local optima and simultaneously augmented the crossover and mutation operators. Maoqing Zhang et al. [26] introduced perturbations to specific parent individuals during the crossover operation to eliminate duplicate individuals, thereby inhibiting premature convergence to local optima. GU X. W. et al. [27] employed a symmetrical Latin hypercube design for the initialization of the population and assimilated the procedures of mutation and crossover from the algorithmic approach of differential evolution with the aim of expediting convergence. Furthermore, they dynamically modulated the control parameters of these operators with the intention of increasing the diversity among the candidates. Z.B. Zhao et al. [28] decomposed the partition of the original population in multiple sub-populations (each is apportioned heterogeneous crossover operators) and introduced a set of dominant solutions to augment algorithm performance. They also exploited the dominant solution set to dynamically regulate the size of each sub-population; consequently, the stability of the algorithm is enhanced. Y.C. Zhu et al. [29] optimized the crossover and mutation operators of the genetic algorithm through formulating an advanced crossover model, effectively circumventing the algorithm from being entrapped in local optima. M.Q. Zhang et al. [30] seamlessly integrated the Lévy distribution within the fast non-dominated sorting genetic algorithm to address the matter of generating an abundance of duplicate individuals ensuing from the tournament selection strategy, thus diminishing population diversity. Z.H. et al. [31] devised an enhanced form of the non-dominated sorting genetic algorithm II (NSGA-II) to optimize the multi-objective task allocation model under constraints. Xu Weiye et al. [32] put forward a task allocation strategy for multi-vehicle systems by integrating the non-dominated sorting genetic algorithm II (NSGA-II) with a variable domain search approach. By incorporating feasibility recovery strategies and the concept of immigrant populations, their method enables real-time task reallocation. The simulation outcomes manifest that the proposed approach showcases remarkable robustness and convergence. Zaiwang Lu et al. [33] grappled with the task allocation conundrum in agricultural multi-robot systems through the implementation of an enhanced non-dominated sorting genetic algorithm (NSGA-II). Through the integration of a mechanism that dynamically modulates the probabilities pertaining to crossover and mutation, their approach enhances the adaptability of the task allocation search process. Yunlong Peng et al. [34] tackled the optimization of multi-agent static task allocation in warehouse scenarios by availing an improved iteration of the non-dominated sorting genetic algorithm (NSGA-II). They advanced a pre-allocation strategy founded on auction for initializing the population, which conspicuously hastened the convergence of the task allocation algorithm.

Through these methods, researchers have made significant progress in improving task allocation efficiency and minimizing conflicts and interferences, thereby providing both theoretical and practical support for the task allocation of automated robotic systems.

## 3. Problem Description and Model Formulation

Despite the considerable progress achieved in the multi-robot task allocation issue within contemporary technologies [35,36,37,38], the efficient assignment and scheduling of robots in the railway sector constantly poses a significant challenge.

In large-scale circumstances, allocating all tasks to a solitary robot usually leads to a considerable amount of time being necessary for completion. Consequently, multi-robot collaboration for task accomplishment has emerged as an inevitable tendency [39]. Failing to account for the specific characteristics of the problem and simply allocating tasks randomly or assigning nearby tasks to the same robot can lead to inefficiencies in the system [40]. Currently, research on task allocation for AOBR in the context of railway track maintenance remains scarce. Therefore, the central challenge of this study is determining the most effective way to allocate tasks in order to optimize the efficiency of multi-robot systems for track bolt maintenance.

### 3.1. Problem Description

**Figure 2:**
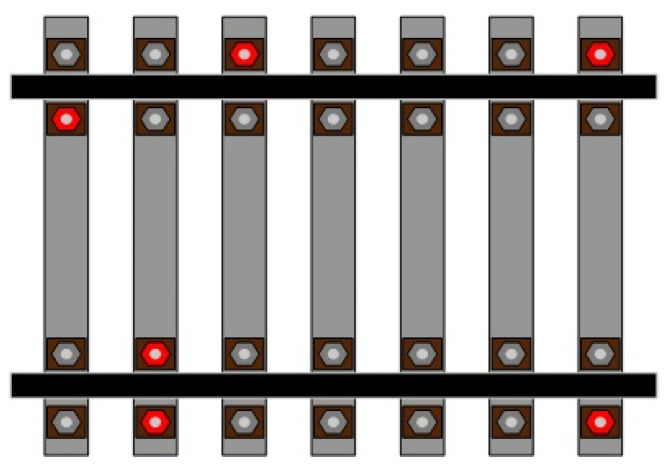
Diagram of partial track bolt maintenance in the window period.

**Figure 3:**
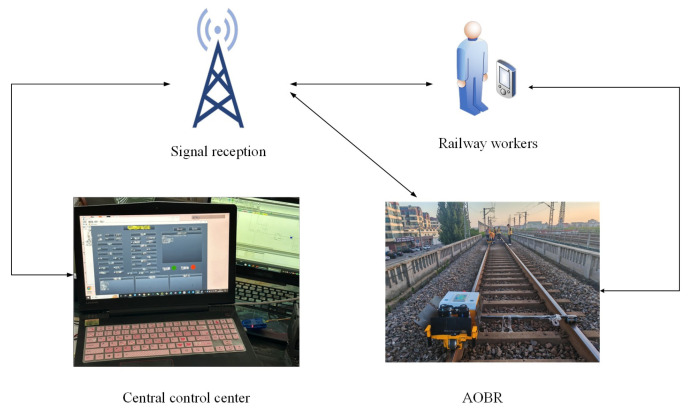
Diagram of task allocation for track bolt maintenance in the window period.

In a track maintenance window, as presented in Figure 2, track bolts need servicing. With the exception of the track replacement operations that necessitate continuous work on the track bolts, all other maintenance tasks are distributed. As depicted in Figure 2, the bolts to be maintained are dispersed and not necessarily concentrated. The gray bolts signify task points that do not require maintenance, while the red bolts indicate task points that need maintenance. As illustrated in Figure 3, the collaborative operation mode of AOBR proceeds as follows. First, the central control center transmits the coordinates of the bolts requiring maintenance to the signal receiving station, which then disseminates the corresponding instructions to the multiple AOBRs scheduled for that day’s maintenance window while concurrently forwarding them to the monitoring screens used by on-site maintenance personnel. The maintenance personnel utilize handheld monitoring devices to issue temporary directives to the AOBRs and to coordinate any AOBRs not yet assigned tasks. Once each AOBR begins operation, it continuously reports its task completion status and device information in real time to both the signal receiving station and the personnel’s monitoring screens. After all AOBRs have completed their respective collaborative tasks, the day’s maintenance work within the designated maintenance window is deemed successfully concluded. All bolts requiring maintenance must be allocated to robots for operation. This study primarily endeavors to determine the optimal task allocation strategy that minimizes both the total travel distance and the maximum completion time of the robots.

In an effort for the purpose of simplifying the problem and to ensure generalization, the subsequent assumptions are posited:

### 3.2. Problem Formulation

The mathematical model presented in this section is expounded as follows:

Parameters:
**Parameters****Meaning**T$T=\{T_{0},T_{1},T_{2},T_{3},\ldots ,T_{n}\}$. The collection of depot and task locations, where $T_{0}$ is the initial entry point to the track, and $T_{1},T_{2},T_{3},\ldots ,T_{n}$ are the *n* task nodes.i,jTask index.$x_{i},y_{i}$The coordinates of the task nodes in the set *T*, i=1,2,3,…,n.$\bar{t_{j}}$The average time a robot takes to maintain each bolt: 5 s.$d_{ij}$Distance from $T_{i}$ to $T_{j}$.$t_{ij}$The travel time for the robot from point $T_{i}$ to point $T_{j}$.R$=\{R_{1},R_{2},R_{3},\ldots ,R_{m}\}$. Set of m robots.kRobot index.$v_{s}$The robot’s travel speed when not performing tasks: 2m/s.$v_{w}$The robot’s travel speed when performing tasks: 45 cm/s.$W_{k}$The completion time for all robots to finish all tasks.$W_{max}$The maximum operation time during the maintenance window.$L_{k}$The sum of the travel distances of all robots upon finishing all tasks.lTask segment index.

Decision variables:
$x_{ik}$If robot $R_{k}$ performs task $T_{i}$, then $x_{ik}=1$; otherwise, $x_{ik}=0$.$y_{ijk}$If robot $R_{k}$ performs task $T_{i}$ after completing task $T_{j}$, then $y_{ijk}=1$; otherwise, $y_{ijk}=0$.

Objective:

(1) $$\min F_{1}=max\sum_{k=1}^{m}W_{k}$$

(2) $$\min F_{2}=\sum_{k=1}^{m}L_{k}$$

Subject to:

(3) $$\sum_{k=1}^{m}x_{ik}=1,\;\forall i\in \{1,2,\ldots ,n\}$$

(4) $$\sum_{i=1}^{n}x_{ik}\geq 1,\;\forall k\in \{1,2,\ldots ,m\}$$

(5) $$x_{0k}=1,\;\forall k\in \{1,2,\ldots ,m\}$$

(6) $$d_{ij}=\left|x_{j}-x_{i}\right|+\left|y_{j}-y_{i}\right|,\;i=0,1,\ldots ,n,\;j=0,1,\ldots ,n$$

(7) $$W_{k}=\sum_{i=1}^{n}\sum_{j=0,j\neq i}^{n}\left(y_{ijk}\cdot \frac{d_{ij}}{v_{w}}\right)+\sum_{j=1}^{n}\left(y_{0jk}\cdot \frac{d_{ij}}{v_{s}}\right)+\sum_{i=0}^{n}\left(x_{ik}\cdot \bar{t_{j}}\right),\;\forall k\in \{1,2,\ldots ,m\}$$

(8) $${\sum_{k=1}^{m}W}_{k}\leq W_{max}$$

(9) $$L_{k}=\sum_{i=0}^{n}\sum_{j=0,j\neq i}^{n}\left(y_{ijk}\cdot d_{ij}\right),\;\forall k\in \{1,2,\ldots ,m\}$$

(10) $$x_{ik}\in \{0,1\},\;\forall i\in \{1,2,\ldots ,n\},\;\forall k\in \{1,2,\ldots ,m\}$$

(11) $$y_{ijk}\in \{0,1\},\;\forall j\in \{1,2,\ldots ,n\},\;\forall k\in \{1,2,\ldots ,m\}$$

Objective function (1) aims to minimize the total completion time for all tasks. Objective function (2) seeks to minimize the total travel distance after all robots have completed their assigned tasks. Constraint (3) affirms that each task is assigned to precisely a single robot. Constraint set (4) ensures that each robot is allocated no less than one task. Constraint (5) manifests that all robots commence from the identical initial entry point. Equation (6) utilizes the Manhattan distance to represent the distance between tasks $T_{i}$ and $T_{j}$. Equation (7) depicts the time requisite for robot $R_{k}$ to accomplish all the assigned tasks. Inequality (8) ensures that the entire time consumed by all robots to accomplish the tasks does not surpass the maximum permissible time within the window period. Equation (9) defines the total travel distance covered by robot $R_{k}$ upon completing all the tasks assigned to it. Finally, constraint sets (10) and (11) specify the valid range or values for the decision variables.

### 3.3. Environment Partitioning and Modeling

In practical operations, the bolts installed on the track are typically uniformly dis-tributed. Nevertheless, the positions of the bolts in need of maintenance tend to be scattered, with task intervals not necessarily uniform. Some areas exhibit a higher density of bolts, while others are sparser. Traditional fixed task allocation schemes encounter difficulties in handling such uneven scenarios, often resulting in some robots being overloaded while others remain idle, thereby adversely affecting the overall operational efficiency. Additionally, existing systems generally lack real-time dynamic adjustment mechanisms. During operations, unexpected circumstances such as robot malfunctions or local environmental changes may arise, and existing systems typically fail to promptly adjust task allocations. This not only leads to a reduction in operational efficiency but also poses risks to both work quality and safety. Moreover, in the context of multi-robot collaboration, existing systems continue to exhibit limitations. The majority of systems rely on independent operational modes and lack effective mechanisms for information sharing and collaboration among robots, thereby limiting overall operational efficiency. To overcome these challenges, there is an urgent need for a task allocation method that comprehensively integrates environmental factors, dynamically adapts to changes in task distribution, and facilitates efficient multi-robot collaboration.

Therefore, this paper proposes a dynamic multi-agent task allocation methodology based on environmental characteristics. This approach enables flexible partitioning of task areas according to the actual distribution of track bolts, facilitates dynamic task allocation, and allows for real-time adjustments while supporting efficient multi-robot collaborative operations.

Based on the extracted work area, the track is regarded as the *x*-axis, and the task bolts to be serviced are considered as points distributed along this axis. The *x*-axis is vertically divided into a number of small sections, each with a length of Δx. The number of sections is determined as shown in Equation (12):

(12) $$a=\frac{L}{\Delta x}$$

In Equation (12), Δx represents the length of the vehicle; Δx=0.9 m; *L* denotes the length of the work zone, $L=x_{max}-x_{min}$, and $x_{min}$ are the minimum and maximum values of the x-coordinates of the track bolts, respectively.

The task density $\rho_{x_{i}}$ of the *l*-th segment is given by Equation (13):

(13) $$\rho_{x_{i}}=\frac{N_{T_{i}}}{\Delta x}$$

In Equation (13), $N_{T_{i}}$ represents the number of tasks in the *l*-th subsegment.

The calculation of whether task $t_{i}$ belongs to the *l*-th subsegment is given by Equation (14):

(14) $$l=[\frac{x_{i}-x_{\min}}{a}]+1$$

In Equation (14), $x_{i}$ denotes the *x*-coordinate of task *i*.

In order to dynamically adjust the region size, a task density variation threshold Δρ needs to be defined. The average task density in the environment is selected, as shown in Equation (15):

(15) $$\Delta \rho =\frac{1}{a}\sum_{i=1}^{n}\left|\rho (x_{i+1})-\rho (x_{i})\right|$$

If the task density variation is minor: $\left|\rho (x_{i+1})-\rho (x_{i})\right|>\Delta x$, the segment is merged with the previous segment, increasing the segment length. The adjusted segment length is denoted as $\Delta x_{i}^{\prime }$, as shown in Equation (16):

(16) $$\Delta x_{i}^{\prime }=\Delta x_{i}+k(\Delta \rho -\left|\rho (x_{i+1})-\rho (x_{i})\right|)$$

In Equation (16), *k* is the adjustment factor that controls the extent of the length increase.

If the task density change is significant, $\left|\rho (x_{i+1})-\rho (x_{i})\right|\geq \Delta x$, the segment will be subdivided, increasing the length of adjacent segments and decreasing the length of the current segment. Let the adjusted segment length be $\Delta x_{i}^{\prime \prime }$, as shown in Equation (17):

(17) $$\Delta x_{i}^{\prime \prime }=\Delta x_{i}+k(\left|\rho (x_{i+1})-\rho (x_{i})\right|-\Delta \rho )$$

In Equation (17), *k* is the adjustment factor that controls the magnitude of the length reduction.

Through the abovementioned dynamic adjustment, a novel set of region lengths $\Delta x_{1}^{\prime },\Delta x_{2}^{\prime },\Delta x_{3}^{\prime },\ldots \Delta x_{o}^{\prime }$ is attained.

These lengths represent the distribution of task densities, i.e., regions with higher task densities are partitioned into more refined segments, while regions with lower task densities are divided into coarser segments.

The total task density $\rho^{\prime }$ within each adjusted region can be calculated as:

(18) $$\rho^{\prime }=\sum_{i=i}^{o}\rho (x_{i})\cdot \Delta x_{i}^{\prime }$$

In Equation (18), *o* represents the number of adjusted segments, and this total is used for subsequent task allocation.

## 4. Methodology

The task allocation problem for AOBRs in track bolt maintenance, which is the central focus of this study, is classified as an NP-hard problem in combinatorial optimization. While genetic algorithms are known for their strong global search capabilities, they are prone to premature convergence and exhibit relatively slow convergence rates. To effectively optimize multi-robot task allocation, this paper proposes an enhanced version of the NSGA-II algorithm. NSGA-II primarily employs non-dominated sorting and crowding distance mechanisms to preserve the diversity of the solution set. However, it tends to exhibit slower convergence rates when addressing complex multi-objective optimization problems. In contrast, MOPSO utilizes a velocity update mechanism based on swarm intelligence to achieve faster exploration but is more prone to becoming trapped in local optima. By integrating NSGA-II and MOPSO, this study aims to balance efficient search space exploration with the maintenance of solution set diversity, thereby generating a more stable and higher quality Pareto front [8]. To maintain solution diversity and preserve elite solutions, a multi-elite archive maintenance strategy is employed. Furthermore, to enhance the refinement of the current optimal solutions during periods of high population diversity, an adaptive mechanism for adjusting crossover and mutation probabilities is introduced. Additionally, with the intent of enhancing the local exploration capacity of the NSGA-II algorithm, this study fuses the MOPSO algorithm with NSGA-II. By adjusting the velocity and position of particles, the algorithm allows better exploration of the search space. When integrated with NSGA-II’s local search ability, this approach strengthens global search capabilities and boosts overall optimization performance.

### 4.1. Fundamentals of NSGA-II Algorithm

Multi-robot task allocation is a classic multi-objective optimization problem, where the primary challenge lies in managing the interactions between multiple objective functions using multi-objective genetic algorithms. The aim is to identify a solution set that optimally maximizes the performance of each objective function, known as the Pareto optimal solution.

NSGA-II (non-dominated sorting genetic algorithm II) is an enhanced version of the original NSGA algorithm, introduced by Deb et al. in 2002 [41]. While NSGA-II follows a procedure similar to that of traditional genetic algorithms, it differs primarily in its selection operator. The core concept of NSGA-II is as follows: The algorithm first performs non-dominated sorting, classifying the population into layers based on dominance and non-dominance relationships among individuals. Subsequently, a tournament selection strategy is employed, where two individuals are randomly chosen from the population for comparison. If both individuals belong to the same front, the one with the greater crowding distance is preferred, as a larger crowding distance indicates greater diversity within the population. If the selected individuals belong to different fronts, the individual with the lower rank value is prioritized, regardless of the crowding distance. This is because, in multi-objective optimization, dominated individuals are considered to have lower priority than those that dominate them.

NSGA-II is widely regarded as one of the most commonly used multi-objective genetic algorithms. It streamlines the complexities associated with non-dominated sorting, offering advantages such as improved computational efficiency and superior convergence properties of the solution set. As a result, it is often used as a benchmark for evaluating multi-objective optimization algorithms. In this study, the NSGA-II algorithm is specifically adapted to address the multi-robot task allocation problem for AOBRs within a railway maintenance context. The overall process of NSGA-II algorithm in AOBR task allocation is as follows.

Step 1: input parameters: *M*, *N*, population size $P_{size}$, crossover probability $P_{c}$, mutation probability $P_{m}$, maximum number of iterations *gen*, task coordinates.

Step 2: Randomly generate the initial population $P_{0}$. The population size is $P_{size}$.

Step 3: calculate the values of the objective function and constraint violations for each individual in the population.

Step 4: non-dominated sorting and crowding distance calculation.

Step 5: use the tournament selection method to select individuals for the parent population $Q_{0}$.

Step 6: perform crossover operations on the parent population $Q_{0}$ to generate the offspring population $R_{0}$, and then apply mutation operations to the offspring population $R_{0}$.

Step 7: Merge the populations: $R_{t}=P_{t}\cup Q_{t}$. The population size is 2 $P_{size}$.

Step 8: perform non-dominated sorting on the merged population $R_{t}$ to obtain multiple non-dominated fronts $F_{1},F_{2},\ldots$.

Step 9: select solutions from the new non-dominated fronts $F_{1},F_{2},\ldots$ starting with $F_{1}$, and iteratively choose solutions until the total number of selected individuals reaches *N*, ensuring dominance in the selection process.

Step 10: if the maximum algebraic generation gen is reached, the algorithm terminates, determine the new population $R_{t}$; otherwise, go back to step 2.

Step 11: output the final population $P_{gen}$.

The algorithm initiates by inputting the number of robots *M*, the number of tasks *N*, the population size $P_{size}$, the crossover probability $P_{c}$, the mutation probability $P_{m}$, and other pertinent parameters. The initial population $P_{0}$ is randomly generated in line with the defined parameters, having a size of $P_{size}$. The crossover and mutation operations are executed on the parent population $Q_{0}$ to generate the offspring population $R_{0}$. The parent population $P_{t}$ and the offspring population $Q_{t}$ are combined to constitute a new population $R_{t}$. A non-dominated sorting is carried out on the merged population $R_{t}$, giving rise to multiple non-dominated fronts $F_{1},F_{2},\ldots$. Starting from the first front $F_{1}$, solutions are successively selected until the number of selected solutions attains $P_{size}$. If the last front $F_{i}$ contains an excessive number of solutions, individuals are selected in accordance with their crowding distance, with priority given to those with a higher crowding distance. The selected solutions are then employed to form the new population $P_{t+1}$. This process is repeated until the maximum number of iterations is attained, at which point, the non-dominated front of the final population is output as the Pareto optimal solution set.

### 4.2. Solution Representation and Encoding

For the sake of simplicity, a direct solution encoding scheme is utilized. This algorithm adopts a real-number encoding format, in conjunction with task-order encoding and separators for task allocation. Suppose that *n* maintenance tasks (represented by bolts) need to be assigned to *m* robots. In this situation, the solution is represented by a vector of length m+n−1. A solution can be depicted as $\alpha =(\mu_{1},0,\mu_{2},0,\ldots 0,\mu_{m})$, where $\mu_{k}=\mu_{k,1},\mu_{k,2},\ldots ,\mu_{k,nk}$, k=1,2,…,m, corresponds to a robot $R_{k}$’s maintenance task sequence, with $n_{k}$ tasks assigned to robot $R_{k}$. The zero value functions as a separator to distinguish the maintenance tasks assigned to adjacent robots. The solution encoding furnishes the sequence through which each robot carries out its assigned tasks.

**Figure 4:**
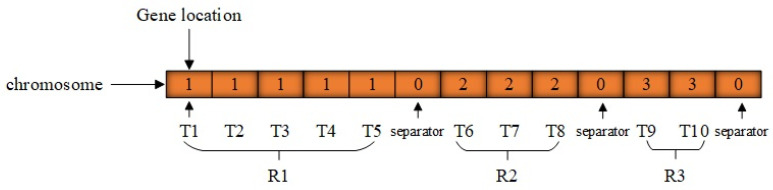
Diagram of chromosome encoding principle.

In each chromosome, the position of the gene denotes a task. Given the specific nature of maintenance tasks, the order of the genes adheres to the task sequence, while the gene values correspond to the robot numbers that will undertake the task. A separator, represented by 0, is utilized to distinguish between adjacent robots. The encoding length is m+n−1, and the chromosome expression based on the encoding design is presented in Figure 4. For instance, if n=10, m=3, based on the solution representation and encoding design, tasks 1–5 are assigned to robot 1, tasks 6–8 to robot 2, and tasks 9–10 to robot 3. Thus, the solution for this example is represented as follows: robots $R_{1}$, $R_{2}$, and $R_{3}$ carry out maintenance tasks according to the sequences $\left(T_{1},T_{2},T_{3},T_{4},T_{5}\right)$, $\left(T_{6},T_{7},T_{8}\right)$, and $\left(T_{9},T_{10}\right)$, respectively. Since the task sequences between adjacent robots are separated by 0, the solution can be depicted as $\alpha =(\mu_{1},0,\mu_{2},0,\mu_{3})$, where $\mu_{1}=(T_{1},T_{2},T_{3},T_{4},T_{5})$, $\mu_{2}=(T_{6},T_{7},T_{8})$, $\mu_{3}=(T_{9},T_{10})$, $n_{1}=5$, $n_{2}=3$, $n_{3}=2$.

### 4.3. Multi-Elite Archive Maintenance

The multi-elite archive is employed to store the non-dominated solutions obtained during each generation of the optimization process. This archive plays a crucial role in preserving the optimal solutions and serves as a reference for subsequent iterations. The pseudocode for the proposed multi-elite archive management is presented in Algorithm 1.
**Algorithm 1:** Multi-Elite Archive
1**Input:** New Population, Multi-Elite Archive *A*, Archive Capacity $N_{A}$, Maximum   Archive Capacity $N_{A}^{\prime }$.2  Initialize the Multi-Elite Archive A=∅.3  In the initial generation of the algorithm, all non-dominated solutions from the   starting population are stored in the archive.4**IF** t < gen **Then**5  Add all non-dominated solutions from the new population $P_{t}$ into the archive   set *A*.6  For each solution $x_{i}$ added to the archive, check whether any other solution   in the archive dominates $x_{i}$.7  **IF**
$x_{i}$ is dominated **Then**8   $x_{i}$ is removed from the archive.9  **ElseIf**
$N_{A}\geq N_{A}^{\prime }$
**Then**
10   Calculate the crowding distance $d_{i}$.11**End If**12**Output:** Elite archive A

At the outset of the algorithm, the multi-elite archive A=∅ is initialized. Initially, non-dominated solutions are selected, and, in the first generation, all non-dominated solutions belonging to the initial population are incorporated into the archive. For a solution $x_{i}$ and $x_{j}$, if solution $x_{i}$ is not inferior to solution $x_{j}$ on all objectives and at least superior on one objective, then $x_{i}$ is deemed to dominate $x_{j}$. Non-dominated sorting is adopted to identify the non-dominated solutions, which are subsequently added to archive *A*. During the optimization process of each generation, the archive is constantly updated with new solutions to uphold the optimal set of non-dominated solutions. After each generation, a new population $R_{t}$ is generated, and all non-dominated solutions from $R_{t}$ are added as candidate solutions to archive *A*. Once new solutions are added, there might exist dominance relationships among the solutions in the archive. Hence, dominated solutions need to be eliminated, and the optimal solutions should be retained via an archive cleaning process. Additionally, if the archive size $N_{A}$ exceeds the maximum capacity $N_{A}^{\prime }$, some solutions must be removed to sustain the archive capacity. With the aim of preserving diversity within the archive, the crowding distance $d_{i}$ of each solution is calculated to evaluate its sparsity within the objective space. Solutions with larger crowding distances are regarded as more sparse and should be given priority for retention. Thereafter, the solutions residing within the archive are ranked in consonance with their crowding distances $d_{i}$, with those having the smallest crowding distances being removed to maintain solution diversity. The crowding distance $d_{i}$ of a solution $x_{i}$ is computed, as shown in Equation (19). When selecting parent individuals for the next generation, some solutions from the multi-elite archive A can be selected to guide the generation of the new population. By exploiting the elite solutions in the archive, the population can be directed towards the Pareto front.

(19) $$di=\sum_{k=1}^{M}\left(\frac{f_{k}(x_{i+1})-f_{k}(x_{i-1})}{f_{k}^{\max}-f_{k}^{\min}}\right)$$

In Equation (19), *M* is the number of objectives; $f_{k}(x_{i+1})$ and $f_{k}(x_{i-1})$ are the objective values of the two neighboring solutions to $x_{i}$ on objective *k*; $f_{k}^{\max}$ and $f_{k}^{\min}$ are the maximum and minimum values of objective *k*.

### 4.4. Adaptive Crossover and Mutation Probability Mechanism

Traditional NSGA-II algorithms typically use fixed probabilities for crossover and mutation. However, in the context of AOBR task allocation, the number of robots and the quantity of bolts requiring maintenance may vary. If the crossover and mutation probabilities are kept constant, this could adversely affect high-quality individuals, ultimately compromising the overall performance of the algorithm. Specifically, if the crossover probability $P_{c}$ is excessively high, it may cause excessive perturbation to the high-quality entities; if it is insufficiently high, the search efficiency could deteriorate. In a similar way, if the mutation probability $P_{m}$ is overly high, the algorithm may devolve into a completely random search, leading to slower convergence, whereas, if it is too low, the diversity of the population will decrease, hindering the creation of new individuals. To effectively retain high-quality individuals, this paper suggests an adaptive adjustment mechanism that dynamically regulates the crossover $P_{c}$ and mutation probabilities $P_{m}$ using a sigmoid function [33], as follows:

(20) $$F=\omega_{1}^{f}\frac{f_{1}-f_{1}^{\min}}{f_{1}^{\max}-f_{1}^{\min}}+\omega_{2}^{f}\frac{f_{2}-f_{2}^{\min}}{f_{2}^{\max}-f_{2}^{\min}}+\cdots +\omega_{k}^{f}\frac{f_{k}-f_{k}^{\min}}{f_{k}^{\max}-f_{k}^{\min}}$$

(21) $$P_{c}=\left\{\begin{array}{cc} P_{c\min}+\frac{1}{1+\exp(\frac{F_{\max}-F^{\prime }}{F_{\max}-F_{avg}})} & ,\;F^{\prime }\geq F_{avg}\\ P_{c\max}\; & ,\;F^{\prime }>F_{avg} \end{array}\right.$$

(22) $$P_{m}=\left\{\begin{array}{cc} P_{m\min}+\frac{1}{1+\exp(\frac{F_{\max}-F^{\prime }}{F_{\max}-F_{avg}})} & ,\;F^{\prime }\geq F_{avg}\\ P_{m\max}\; & ,\;F^{\prime }>F_{avg} \end{array}\right.$$

In Equation (20), *F* represents the defined weight function, which normalizes the objective function $f_{k}$ and sets the corresponding weight $\omega_{k}^{f}$. In Equations (21) and (22), $P_{c\min}$ and $P_{c\max}$ represent the minimum and maximum crossover probabilities, separately, while $P_{m\min}$ and $P_{m\max}$ represent the minimum and maximum mutation probabilities, respectively. $F^{\prime }$ corresponds to the individual with the highest fitness value. $F_{avg}$ represents the average fitness value of the individuals in the current population. When the difference $F_{\max}-F_{avg}$ diminishes, the maximum fitness value of the population converges toward the average fitness value of the population. At this point, the algorithm could either converge to the global optimum or become stuck in a local optimum. To maintain population diversity, the crossover probability $P_{c}$ and the mutation probability $P_{m}$ are augmented. Conversely, when $F_{\max}-F_{avg}$ increases, the algorithm conducts the opposite operations to retain high-quality individuals and eliminate inferior ones.

### 4.5. Fusion of MOPSO and NSGA-II Algorithms

The NSGA-II algorithm excels at refining the search within local regions by continuously generating new candidate solutions through crossover and mutation operations, thereby driving solutions closer to the Pareto front. Its elite preservation mechanism ensures that high-quality solutions are retained in each generation, providing a robust starting point and a solid foundation for the search process. Conversely, MOPSO is a multi-objective optimization approach derived from the particle swarm optimization (PSO) algorithm. It is widely applied in fields such as engineering design and resource allocation. MOPSO aims to determine the Pareto optimal set by mimicking the social behavior of a particle swarm, excelling particularly in global exploration. By updating particle positions and velocities, the swarm can comprehensively explore the entire solution space, covering regions that have not been adequately explored, thus manifesting a strong global search capability [42]. In PSO, each particle adjusts its direction and velocity based on its previous trajectory and the information shared within the swarm, reflecting the principles of swarm intelligence.

In particle swarm optimization, each particle adjusts its direction and velocity for the subsequent movement based on its own historical flight trajectory and the information shared within the swarm, thereby reflecting the characteristics of swarm intelligence. The particle update equations are provided by Equations (23) and (24).

(23) $$v_{i+1}=\omega v_{i}+c_{1}r_{1}(p_{best}-x_{i})+c_{2}r_{2}(g_{best}-x_{i})$$

(24) $$x_{i+1}=x_{i}+v_{i+1}$$

In Equations (23) and (24), ω is the inertia weight; $c_{1}$ and $c_{2}$ are the cognitive and social learning factors; $r_{1}$ and $r_{2}$ are random numbers; $p_{best}$ represents the particle’s own historical best position; $g_{best}$ is the global best position within the multi-elite archive.

**Figure 5:**
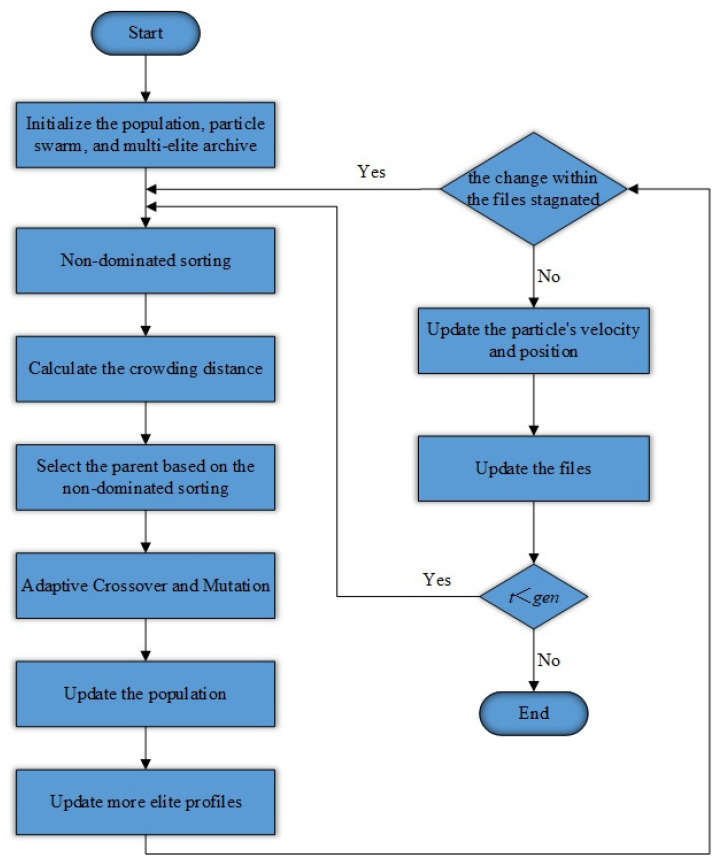
Flowchart exemplifying the enhanced NSGA-II integration algorithm.

In the incipient stages of optimization, NSGA-II perpetually generates locally optimal solutions within the solution space by means of crossover and mutation operations, thereby establishing the initial Pareto front. Subsequently, the MOPSO algorithm is employed for global exploration, steering the solutions towards better directions by updating the particles’ velocities and positions. Therefore, the integration of these two algorithms is aimed at capitalizing on the results of local optimization and, via global exploration, overcoming local optima to further elevate the overall solution quality. The flowchart of the enhanced NSGA-II integration algorithm is manifested in Figure 5.

In the early stages of the algorithm, NSGA-II promotes information exchange within the population through crossover and mutation operations, quickly generating the initial Pareto front. This phase focuses on population growth, utilizing non-dominated sorting and elite retention to obtain a diverse distribution of initial non-dominated solutions while preserving sufficient diversity in the solution space. The primary goal of this process is to build a high-quality initial solution set that adequately represents the problem’s solution space.

To enhance the algorithm’s coverage, a multi-elite archive mechanism is incorporated to document the non-dominated solutions from each iteration of both NSGA-II and MOPSO, dynamically updating the archive contents to guarantee that it covers a wider range of the Pareto front. After the NSGA-II operations are accomplished, the multi-elite archive stores the current optimal solutions, offering a reference for the global optimal solutions during the subsequent MOPSO phase. Through this mechanism, the algorithm can exploit the information in the archive to guide particles towards the current optimal regions, thereby enhancing the optimization performance.

In the subsequent iterations, MOPSO undertakes a large-scale exploration of the solution space through the velocity update mechanism of the particle swarm, uncovering potential high-quality solutions that NSGA-II might have missed. This alternating strategy enables the algorithm to undertake a more extensive exploration of the solution space, starting from the initial high-quality solution set and determining additional optimal solutions.

The crossover and mutation operations of NSGA-II predominantly center on local searches, whereas MOPSO accentuates global searches. By availing the potent global search capacity of MOPSO, the particle swarm can progress towards superior solutions, thereby further elevating the overall quality of the Pareto front. Therefore, combining MOPSO with NSGA-II not only effectively increases population diversity but also helps the algorithm avoid local optima, greatly improving its convergence performance.

This hybrid strategy is designed to establish a high-quality initial solution set for the problem during the initial development stage of NSGA-II. Thereafter, by exploiting the global exploration potential of MOPSO, the solutions are guided towards more promising domains, augmenting the coverage of the solution space and elevating the overall performance of the Pareto front. By alternating between the NSGA-II and MOPSO phases, the algorithm effectively leverages the strengths of both approaches, balancing local refinement with global exploration, speeding up convergence, and utilizing the optimal solutions in the multi-elite archive to guide the search process.

The alternating optimization process guarantees the construction of a robust initial Pareto front during the early stages of the algorithm. In subsequent phases, it further expands the solution space through a global search, identifying additional high-quality solutions. This approach thus results in superior multi-objective optimization performance. NSGA-II initially provides a strong solution set, establishing a solid foundation for further exploration of the solution space. Simultaneously, MOPSO, with its global search capability, enables the population to escape local optima, covering a broader region of the Pareto front. This not only enhances solution quality but also increases diversity, ensuring the overall success of the optimization process.

## 5. Simulation Experiment Results and Analysis

### 5.1. Simulation Experiment Parameter Settings

This section will simulate the track bolt maintenance scenario based on real-world railway maintenance windows. The number of maintenance tasks is identified as n={30,50,70}. Since the track bolts are installed in accordance with a standard assembly on both sides of the track, with a fixed spacing of 0.6 m between adjacent bolts on the same side, the coordinates of the bolts will be randomly distributed to reflect the actual maintenance requirements. The quantity of maintenance robots is set to be m={3,5,7,10}. All experiments are carried out and validated on the MATLAB platform. Experimental parameter settings: maximum number of iterations *gen* = 1000, population size $P_{size}$ = 100, $N_{A}^{\prime }$ = 100, each group repeats the experiment 5 times.

### 5.2. Evaluation Metrics

#### 5.2.1. HV

HV (hypervolume) is defined as the quantity of the objective space occupied by the individuals within the solution set, delimited by a reference mark [43]. The HV formula is shown in Equation (25). In general, a higher HV value implies a superior overall performance of the algorithm.

(25) $$HV(P)=\delta (\cup_{i=x}^{\left|P\right|}v_{x})$$

In Equation (25), δ represents the Lebesgue measure for volume calculation; *P* denotes the reference point; $\left|P\right|$ denotes the count of non-dominated solutions; $v_{x}$ represents the hypervolume generated by the *x*-th solution, the reference point, and the solution set.

#### 5.2.2. IGD

IGD (inverted generational distance) is utilized to evaluate both the convergence and diversity of the non-dominated solution collection [44]. The calculation formula of this metric is displayed in Equation (26). It gauges the performance of the algorithm through ascertaining the mean interval between the individuals in the non-dominated solution set produced by the algorithm and the true Pareto optimal set. Generally, a lower IGD value suggests a more outstanding overall performance of the algorithm.

(26) $$IGD(P,P^{*})=\frac{\sum_{x\in P^{*}}d(x,P)}{\left|P^{*}\right|}$$

In Equation (26), $P^{*}$ denotes the non-dominated solution set obtained by running each algorithm for each test independently five times; d(x,P) represents the Euclidean distance between a point x in set $P^{*}$ and the closest point in set *P*.

### 5.3. Analysis of Simulation Experiment Results

**Figure 6:**
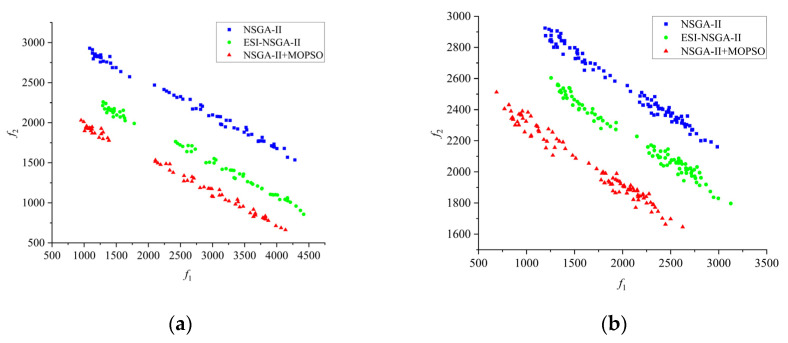

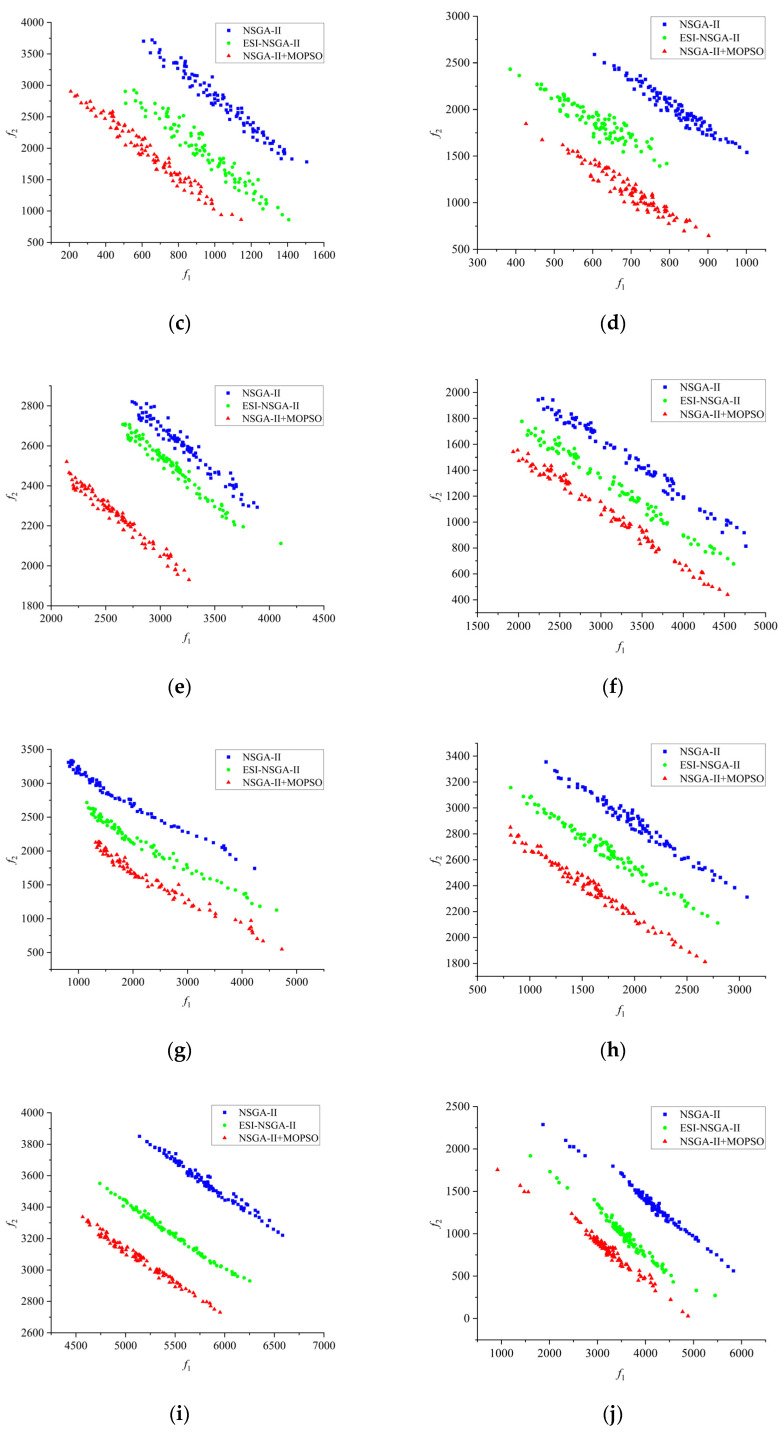

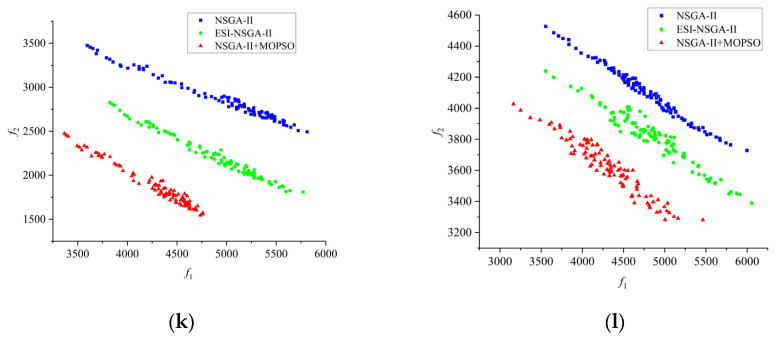
Pareto front of the algorithms. (**a**) n=30,m=3; (**b**) n=30,m=5; (**c**) n=30,m=7; (**d**) n=30,m=10; (**e**) n=50,m=3; (**f**) n=50,m=5; (**g**) n=50,m=7; (**h**) n=50,m=10; (**i**) n=70,m=3; (**j**) n=70,m=5; (**k**) n=70,m=7; (**l**) n=70,m=10.

A simulation was conducted in MATLAB 2022b using a real-world railway maintenance window scenario for track bolt maintenance. The number of maintenance tasks and robots was configured according to actual maintenance needs. Following the task allocation, Figure 6 illustrates the distribution of non-dominated solutions produced by various algorithms in the objective space.

Figure 6 presents 12 Pareto front graphs. As shown in Figure 6, compared with the Pareto front generated by the NSGA-II algorithm, the Pareto front obtained by the improved NSGA-II hybrid algorithm is positioned closer to the lower-left corner of the graph. This finding indicates that, in most cases, the improved algorithm can dominate the solutions produced by other comparative algorithms. For the current problem, the improved algorithm is capable of identifying a more preferable set of solutions. To make an objective comparison of the performance of the various algorithms, the HV and IGD values for each algorithm were computed, and the results are presented in Table 1 and Table 2.

**Figure 7:**
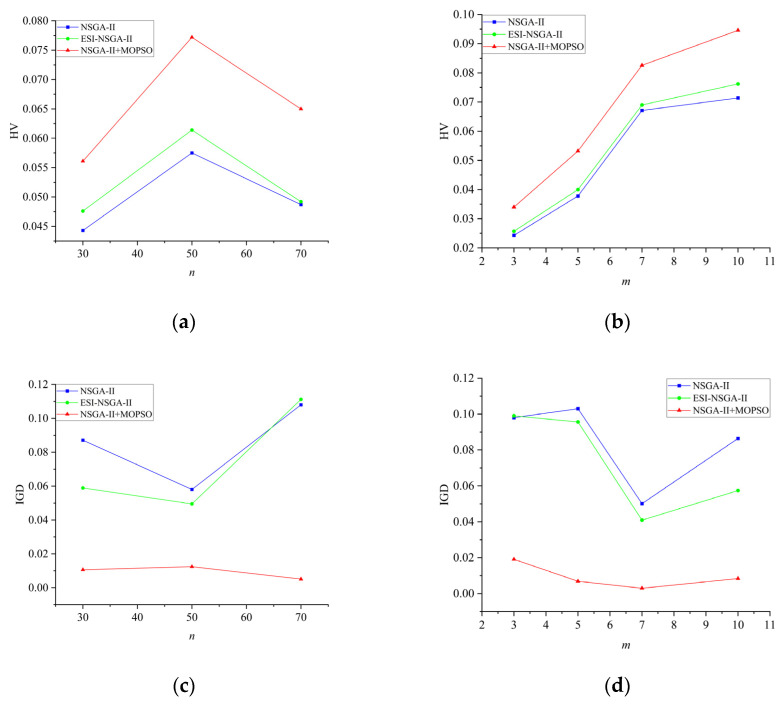
Interaction plot of all algorithms and example size (**a**) *n* and algorithm (HV); (**b**) *m* and algorithm (HV); (**c**) *n* and algorithm (IGD); (**d**) *m* and algorithm (IGD).

We undertook comparative experiments involving three algorithms: NSGA-II, ESI-NSGA-II, and NSGA-II + MOPSO. Each algorithm was independently executed five times for each test instance. Table 1 and Table 2 furnish the average HV and IGD values for each algorithm under the identical task number *n* and robot number *m*. As observed from the results in Table 1 and Table 2, the improved NSGA-II + MOPSO hybrid algorithm shows an increase in the average HV value by 26.64%, 34.26%, and 33.47%, and an improvement in the average IGD value by 87.83%, 78.62%, and 95.28% for different task and AOBR quantities, respectively. These values outperform the corresponding averages obtained using the NSGA-II algorithm, demonstrating superior overall performance. To intuitively illustrate the distribution of solutions generated by the improved algorithm, interaction graphs for each algorithm were constructed under the HV and IGD metrics with task number *n* and robot number *m*, as shown in Figure 7.

As illustrated in Figure 7, under the HV metric, irrespective of the problem size, the performance of the improved NSGA-II + MOPSO fusion algorithm accords with the trend witnessed in the HV metric. Notably, the IGD values of the improved algorithm evince less fluctuation, significantly outstripping the other algorithms. Furthermore, as the quantity of robots ascends, the performance undergoes an enhancement. This indicates that the proposed improved NSGA-II + MOPSO hybrid algorithm demonstrates superior efficiency and stability in optimization performance.

**Figure 8:**
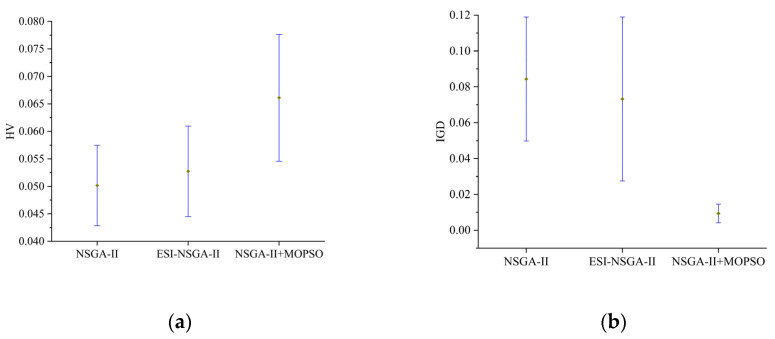
Average value chart of all algorithms. (**a**) HV; (**b**) IGD.

Figure 8 presents the mean HV and IGD values of the three algorithms within a 95% confidence interval. It is evident that the enhanced NSGA-II + MOPSO hybrid algorithm achieves a significantly higher average HV value compared with the other two algorithms, while its average IGD value is notably lower. Furthermore, the confidence intervals for both HV and IGD metrics of the enhanced NSGA-II + MOPSO hybrid algorithm do not overlap with those of the other two algorithms, highlighting a significant performance difference. Therefore, it can be concluded that the enhanced NSGA-II + MOPSO hybrid algorithm outperforms the other algorithms in solving the multi-robot task allocation problem investigated in this study.

## 6. Conclusions

This paper presents the application of the NSGA-II + MOPSO hybrid algorithm to optimize multi-agent AOBR task allocation in the context of railway bolt maintenance. To address the multi-robot task allocation problem, an efficient improved NSGA-II + MOPSO hybrid algorithm is proposed. Extensive experimental comparisons with the traditional NSGA-II algorithm and a multi-elite strategy-enhanced NSGA-II algorithm validate the effectiveness and superior performance of the proposed algorithm. This study offers an efficient resolution for the task allocation requisites of AOBR in railway scenarios.

In future research, we will focus on addressing the practical requirements of AOBR collaborative control through the following improvements. First, we aim to further optimize multi-robot collaborative task allocation strategies to enhance the algorithm’s adaptability to diverse operational conditions and its effectiveness in complex multi-constrained scenarios. Second, we will explore and integrate advanced collaborative control strategies, such as reinforcement learning and multi-agent distributed decision-making mechanisms, to improve the autonomy and fault tolerance of the robotic systems. Finally, by incorporating fault detection and diagnosis techniques alongside real-time scheduling and resource management strategies, we seek to further enhance the algorithm’s stability and robustness in non-ideal working environments, thereby meeting the high reliability and efficiency demands of railway bolt maintenance operations.

## Figures and Tables

**Table 1:** HV values of the algorithms.

n×m	NSGA-II	ESI-NSGA-II	NSGA-II + MOPSO
30×3	0.0306	0.0391	0.0403
30×5	0.0593	0.0576	0.0761
30×7	0.0681	0.0643	0.0749
30×10	0.0193	0.0294	0.0329
Mean	0.0443	0.0476	0.0561
50×3	0.0109	0.0119	0.0179
50×5	0.0361	0.0439	0.0543
50×7	0.0794	0.0836	0.1061
50×10	0.1035	0.1061	0.1306
Mean	0.0575	0.0614	0.0772
70×3	0.0314	0.0261	0.0437
70×5	0.0181	0.0184	0.0291
70×7	0.0538	0.0591	0.0669
70×10	0.0914	0.0932	0.1204
Mean	0.0487	0.0492	0.0650

**Table 2:** IGD values of the algorithms.

n×m	NSGA-II	ESI-NSGA-II	NSGA-II + MOPSO
30×3	0.0861	0.0563	0.0153
30×5	0.0531	0.0439	0.0039
30×7	0.0294	0.0216	0.0016
30×10	0.1799	0.1137	0.0217
Mean	0.0871	0.0589	0.0106
50×3	0.0862	0.0793	0.0392
50×5	0.0615	0.0594	0.0032
50×7	0.0461	0.0327	0.0049
50×10	0.0383	0.0267	0.0021
Mean	0.0580	0.0495	0.0124
70×3	0.1218	0.1613	0.0027
70×5	0.1943	0.1834	0.0135
70×7	0.0749	0.0683	0.0026
70×10	0.0411	0.0319	0.0015
Mean	0.1080	0.1112	0.0051

## Data Availability

Data are contained within the article.
